# Edge states in plasmonic meta-arrays

**DOI:** 10.1515/nanoph-2022-0258

**Published:** 2022-07-07

**Authors:** Qiuchen Yan, En Cao, Xiaoyong Hu, Zhuochen Du, Yutian Ao, Saisai Chu, Quan Sun, Xu Shi, C. T. Chan, Qihuang Gong, Hiroaki Misawa

**Affiliations:** State Key Laboratory for Mesoscopic Physics and Department of Physics, Collaborative Innovation Center of Quantum Matter & Frontiers Science Center for Nano-optoelectronics, Beijing Academy of Quantum Information Sciences, Peking University, Beijing 100871, P. R. China; Research Institute for Electronic Science, Hokkaido University, Sapporo 001-0021, Japan; Peking University Yangtze Delta Institute of Optoelectronics, Nantong, Jiangsu 226010, P. R. China; Collaborative Innovation Center of Extreme Optics, Shanxi University, Taiyuan, Shanxi 030006, P. R. China; Creative Research Institution, Hokkaido University, Sapporo, 001-0021, Japan; Department of Physics and Institute for Advanced Study, Hong Kong University of Science and Technology, Clear Water Bay, Kowloon, Hong Kong, China; Center for Emergent Functional Matter Science, National Yang Ming Chiao Tung University, Hsinchu 30010, Taiwan

**Keywords:** edge state, lasers, nanoscale, photoemission electron microscopy, plasmonic array, quantum entanglement

## Abstract

Photonic edge states provide a novel platform to control and enhance light–matter interactions. Recently, it becomes increasing popular to generate such localized states using the bulk-edge correspondence of topological photonic crystals. While the topological approach is elegant, the design and fabrication of these complex photonic topological crystals is tedious. Here, we report a simple and effective strategy to construct and steer photonic edge state in a plasmonic meta-array, which just requires a small number of plasmonic nanoparticles to form a simple lattice. To demonstrate the idea, different lattice configurations, including square, triangular, and honeycomb lattices of meta-arrays, are fabricated and measured by using an ultrahigh spatial resolution photoemission electron microscopy. The properties of edge states depend on the geometric details such as the row and column number of the lattice, as well as the gap distance between the particles. Moreover, numerical simulations show that the excited edge states can be used for the generation of the quantum entanglement. This work not only provides a new platform for the study of nanoscale photonic devices, but also open a new way for the fundamental study of nanophotonics based on edge states.

## 1 Introduction

Edge state is an important concept in the physical systems. The responses of a system to the external environment are usually dominated by the system edges which are the boundary between the bulk and the external world and as such, edge modes have special importance. Their transport is characterized by small total loss, strong field enhancement, and it is usually easier to regulate its coupling with other systems. The study of edge states has a long history in the field of surface science, and there have been reported different methods to realize edge modes. The most mathematically elegant way to create edge states is to use topological materials [1–4], with the advantages of the robustness, anti-backscattering and low loss transmission. In photonics, there are a large number of theoretical [5–8] and experimental [9–13] researches to support the edge states in topological systems by using dielectric [9, 14, 15], metal [16–23] and gain materials [24–29] in different dimensions [30–33]. The edge states in nonlinear optics [34–38], non-Hermitian systems [39–42], and the coupling systems between plasmonics and graphene [43–46] can also be realized through topological metasurfaces. However, topological photonic materials usually have complex structures and they generally require large areas in order to have a well-defined bulk, which bring challenges to the sample fabrication and the reduction of integrated chips. It also requires typical excitation methods and external conditions, such as magneto-optical materials and quantum gain materials which need to be excited under the magnetic field. Edge modes of course do not require a topological reason to exist. It is well known that many truncated photonic crystals carry edge states [47–53], but this kind of edge states have specific requirements for the truncated boundary configurations. In addition, some heterostructures can also exhibit edge states where the localized modes appear. However, the design and fabrication of the heterostructures are difficult because of the splicing the multiple different structures or materials [54–56]. Consequently, it is highly desirable for practical applications if compact nanostructures that are easy to fabricate can support edge resonances that can be easily excited [20, 57], [58], [59], [60], [61]. Fortunately, plasmonic nanoarrays can have all the above advantages. Both 1D and 2D plasmonic crystal structures have been studied and used as highly transmissive polarization-independent subtractive color filters [62], nanolasers [63–67] and molecular emission enhancement device [68–70], as well as the devices in quantum systems [70–75]. Compact plasmonic nanoarrays have the potential to support edge states, and these structures are easy to fabricate and be excited, as well as to be integrated.

Here, we report a strategy to construct and steer photonic edge eigenmodes in a structurally ordered or random plasmonic meta-array with a compact size under linear polarized-incident excitation. The system is flexible to design and easy to fabricate. This kind of photonic edge states exist in fairly small plasmonic clusters and are hence different from the topological edge states which depend on a system big enough to define bulk topological invariants. Our system has two edges that are parallel to the polarization direction of optical-localized states, and the interior excitation is suppressed with no optical response. The cluster is compact and the row or column numbers usually do not exceed 4, which means that band theory is not relevant in our studies. Moreover, the lattice constant of this plasmonic meta-array requires no more than twice the lattice unit diameter. That means the gaps between each plasmonic particles are extremely small, and the suppression effect of the interior is strong. These two factors ensure the appearance of edge eigenmodes in the plasmonic meta-arrays under linear polarized-incident excitation. If the polarization direction of the incident light is changed, the excited edge-states positions of the meta-arrays are also changed. It is robust in the sense that the edge state still exists even when one of the edge disks is missing. Furthermore, by analyzing the dephasing time of these edge states in gradual-changed lattice numbers of meta-arrays, it is found that the dephasing time will increase with the decrease of lattice constant, and with the increase of the lattice number that are parallel to the polarized direction. This phenomenon confirms the above required two factors. Plasmonic meta-arrays with different underlying lattice structures, including square, triangular, and honeycomb lattices, can all support edge states that are all verified through experiments by using an ultrahigh spatial resolution photoemission electron microscopy (PEEM), which indicates that our strategy is generally applicable. PEEM instruments can help us measure the plasmonic hot spots and confirm the spatial positions of the measured structures at the same time. This is also a noncontact measurement, which can show the responses of the structure itself to the external light field without disturbance or interference. Last but not least, simulations also indicate that the excited edge state can be an entangled-quantum emitter, which shows that the meta-array can be used not only in the classical but also the quantum applications. This work not only provides a new platform for the study of nanoscale photonic devices based on edge states of eigenmodes, but also open a new way for the fundamental study in nanophotonics.

**Figure 1:**
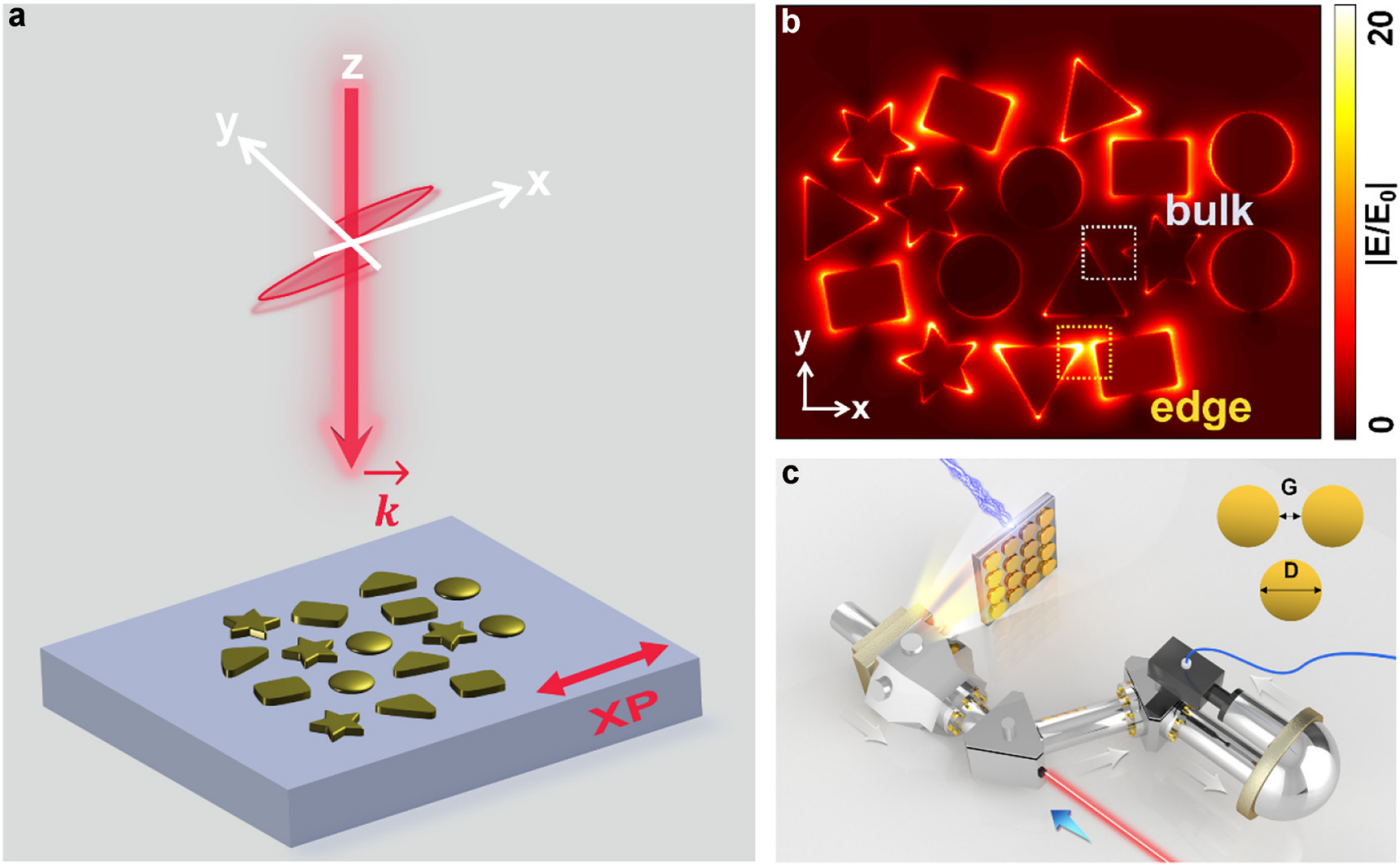
Schematic illustration of random plasmonic meta-array and the experimental setup. (a) Picture of a random array of plasmonic particles with different shapes and gaps between each of particles. The direction of the red arrow, along the *z*-axis, indicates the propagation direction of linear polarized incident plane-wave. The two-way red arrow indicates the direction of XP light. (b) Simulated electric field distribution under XP excitation (by FDTD method). The yellow dashed rectangle represents part of the area of edge and the grey dashed rectangle represents part of the interior. The simulated suppression ratio is 27.7. (c) Schematics of the experimental instruments of PEEM. The blue arrow indicates the direction of the incident laser and the grey arrows indicate the direction of collected electrons escaping from the sample surface. The sample composed of gold disks is fabricated by EBL on the ITO substrate. The gaps G between the nearest disks are 20 nm, and the diameter of each disk is fixed 120 nm in our experiments.

In order to show the simplicity of the designed structures, we first consider a random cluster consisting of plasmonic units of different shapes and gaps between every two units as shown pictorially in Figure 1(a). The direction of red arrow along the *z* axis indicates the propagation direction of the linear polarized incident plane-wave, normally and uniformly exciting the whole cluster. The polarization of the incident light is parallel to the axis *x* (XP), as indicated by a two-way arrow. The simulated electric field distribution under XP excitation by finite-difference time-domain method (Lumerical FDTD Solutions) is shown in Figure 1(b). The upper and lower edges, parallel to the incident-polarized direction, have obvious optical-field enhancement, and the optical responses of the internal units are small. In order to accurately describe the suppression of excitation in the interior, we define a suppression ratio as the ratio of the near-field enhancement density of region produced by the edge units to the near-field enhancement density of region produced by the bulk units. The higher the suppression ratio value, the better the suppression effect. In Figure 1(b), the yellow dashed rectangle represents a part of the edge and the grey dashed rectangle represents an area in the bulk. In this case, the suppression ratio is 27.7 at the excited wavelength of 720 nm, indicating that there are eigenmodes localized near the edge in this random meta-array. The detailed suppression ratio calculation method is given in Supplementary Materials (SM) I.

## 2 Analysis of C4R4 and C5R4 arrays

**Figure 2:**
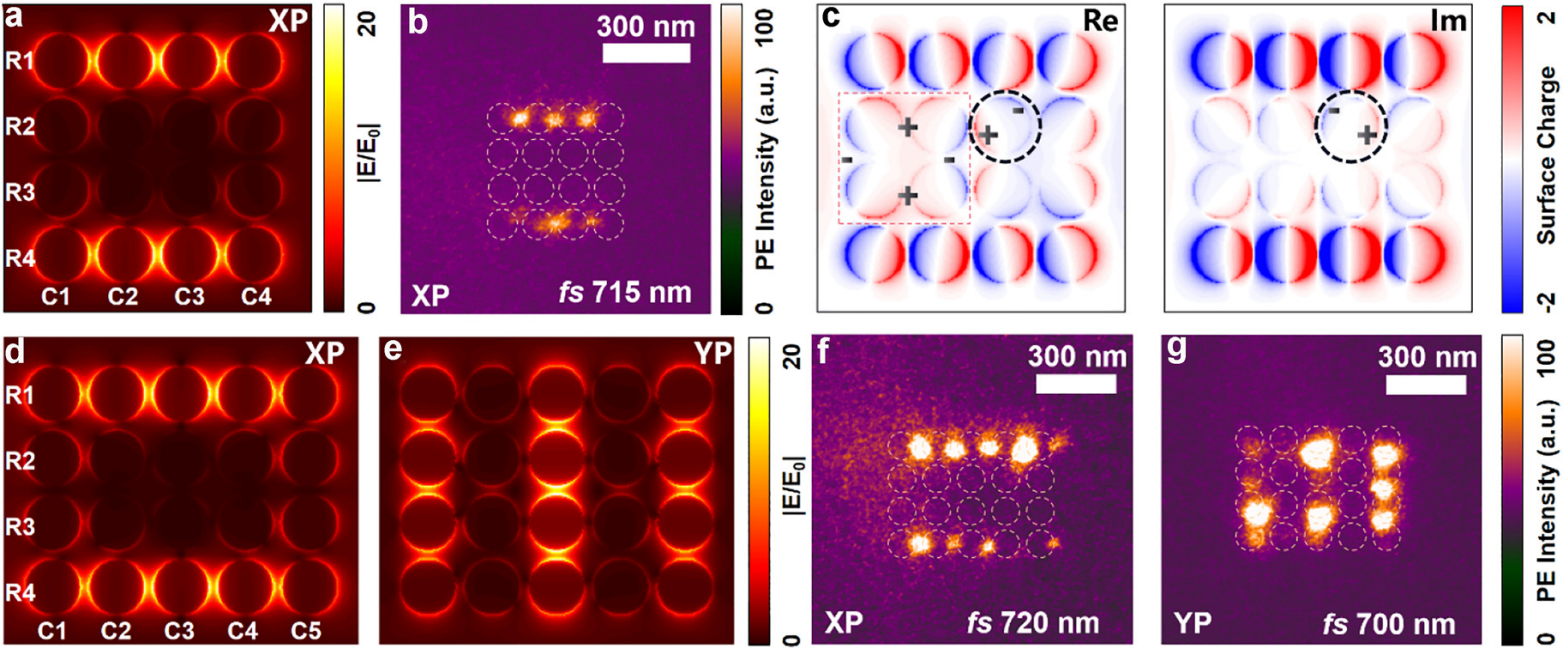
Near-field images of square-lattice simulated and experimental results. (a) Simulated electric field distribution of C4R4 meta-arrays under XP excitation. The columns and rows are number by C1 to C4 and R1 to R4. (b) The experimental near-field image of the edge state in C4R4 meta-arrays under XP excitation. The field of view is 5 um and the excitation wavelength is 715 nm. (c) Simulated real and imaginary parts of surface charge distributions of edge state for C4R4 meta-arrays under XP incidence. The dashed red rectangle marks the large quadrupole mode excitation of the four interior disks. The dashed black circles show the dipole-mode direction is not completely parallel to the polarization direction. (d, e) Simulated electric field distributions under XP and YP excitation for C5R4 arrays. (f, g). Corresponding experimental near-field imaging of edge state and bulk state under XP and YP excitation. The field of view is 5 um and the excitation wavelength is 720 nm and 700 nm, respectively.

In order to analyze this type of edge state that can be found easily in a small cluster, we consider a simple and ordered structure. While we select square lattices because they are convenient to fabricate and to do experiments, other lattice types and configurations can also illustrate this phenomenon, which will be discussed in detail later. As shown in Figure 1(c), the samples are all measured by PEEM instruments with ultra-high time and spatial resolution to obtain the near-field mode distributions. The blue arrow indicates the direction of the incident femtosecond laser with pulse width of 100 fs and the grey arrows indicate the collected direction of electrons escaping from the sample surface. The plasmonic meta-array is constructed by a square lattice of gold disks with a fixed diameter D of 120 nm and thickness of 30 nm. The lattice constant is 140 nm, as a consequence, the gaps G between every two nearest disks are 20 nm which are extremely small. A series of plasmonic meta-arrays with different column numbers, row numbers and lattice types are fabricated by using electron beam lithography (EBL) on the indium tin oxide (ITO) substrates, and gold disk are grown by using helicon sputtering. Taking the meta-arrays with column (C) number of 4 and row (R) number of 4 (C4R4) as an example, an edge state is excited under XP excitation. The simulated electric field distribution of C4R4 array is shown in Figure 2(a), and the experimental near-field image of the edge state is shown in Figure 2(b). In this case, only the edge disks along the polarized direction of incident light have plasmonic resonance responses, that is, the rows R1 and R4, while the plasmonic resonance responses are suppressed in the rows R2 and R3. The experimental field of view is 5 um and its excitation wavelength is 715 nm which is at the meta-array plasmonic resonant peak wavelength. In addition, the suppression ratio is calculated in simulation as 41.46 and in experiment as 6.14. The difference comes from the limitation of experimental conditions, such as the photoemission from the ITO substrates and the scattering light from other excited defect points.

Furthermore, the simulated real and imaginary parts of surface charge distributions of the edge state in trivial plasmonic C4R4 meta-arrays under XP excitation are also analyzed as shown in Figure 2(c), same as the experimental configurations. It is clear that in the real part of the surface charge distributions, the disks in the rows of R1 and R4 show the dipole mode and there is strong coupling strength among these disks and the dipoles of each disk oscillates in phase along the edge. In the rows R2 and R3, each disk exhibits a dipole mode, but neighboring disks have the opposite charge distributions. In the interior, the excited charge of every four disks exhibits a large quadrupole mode that is marked by dashed red rectangle; we call this the “high-order-like” mode. In the imaginary part of the surface charge distributions, the intensities on the rows R1 and R4 are higher than the interior, and every disk shows a dipole mode. If we carefully observe the surface charge distributions of the real and imaginary parts of this whole cluster, nearly every disk has an oblique dipole mode, and the dipole-mode direction is not completely parallel to the polarization direction of incident light as marked by dashed black circles in Figure 2(c). Therefore, the dipole moment of a disk should be calculated by multiplying the polarizability by composing the total electric fields induced by other dipoles in these plasmonic meta-arrays, and the *x*- and *y*-polarized modes are coupled.

The edge states also exist in the meta-arrays with column number of 5 and row number of 4 (C5R4) under XP excitation. The simulated electric field distribution and experimental near-field image are shown in Figure 2(d), (f). The rows of R1 and R4 have plasmonic resonance response, while the response is very weak in rows R2 and R3. The experimental field of view is 5 um and its excitation wavelength is 720 nm at the resonant peak wavelength. The suppression ratio is calculated in simulation as 82.68 and in experiment as 6.21. However, when the polarization direction of incident light is rotated by 90° (the polarized light parallel to the axis *y* (YP) excitation), the edge states on the R1 and R4 cannot be excited completely and the disks in the central column is strongly excited as shown in Figure 2(e), (g). In this case, the columns of C1, C3, and C5 have the plasmonic resonance responses, while they are suppressed in the columns of C2 and C4. The experimental field of view is 5 um and its resonant peak wavelength is 700 nm which has a blue shift than that under XP excitation. The surface charge distributions of C5R4 meta-arrays under XP and YP excitation are also provided in the SM II, the quadrupole mode of disks appear as predicted in the C4R4 meta-array. We infer that the eigenmodes that are localized in the interior of the cluster have charge distributions that is like quadruple modes, and hence they cannot be excited easily using plane wave excitation at normal incidence.

**Figure 3:**
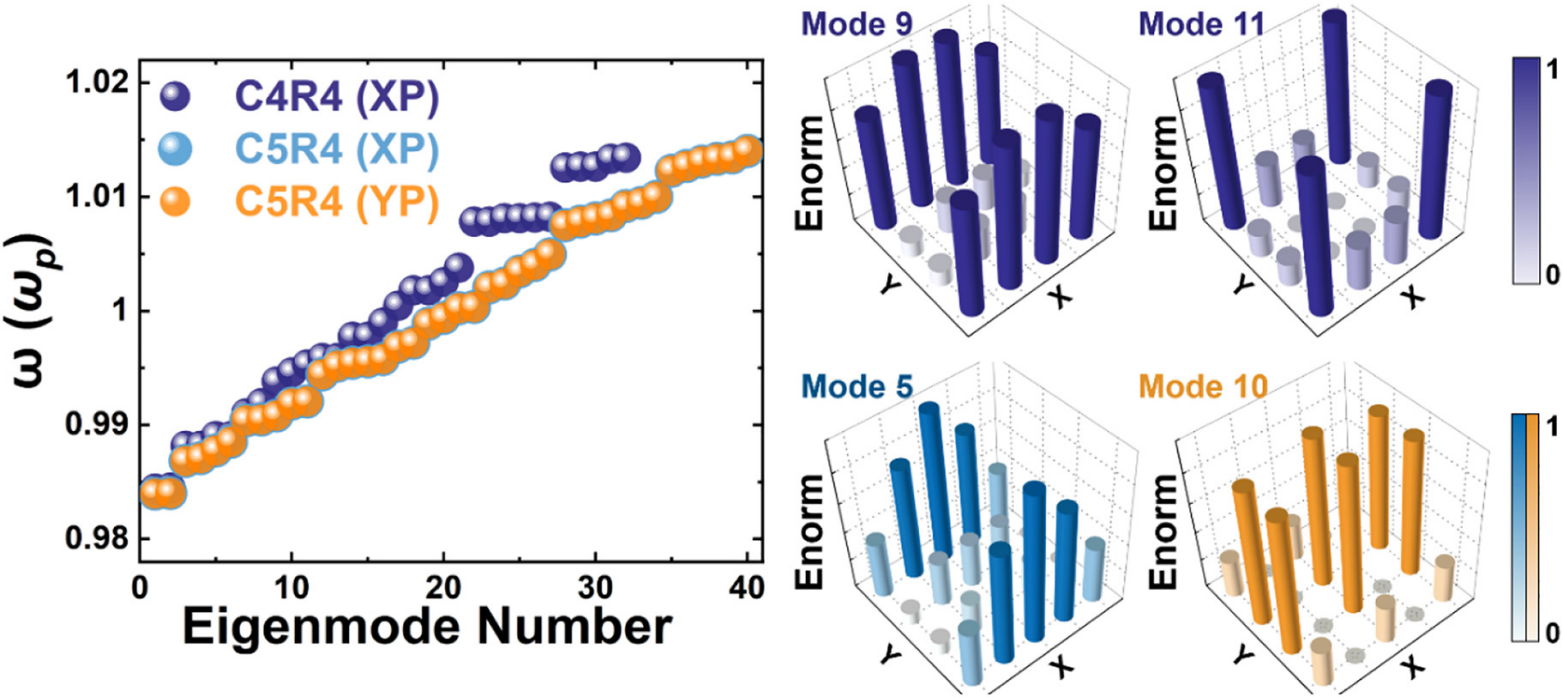
Calculated the eigenvalues of the C4R4 (purples) and C4R5 (blues and oranges) arrays under XP and YP excitation using the model in the text. The normalized electric field distribution of edge state (mode 9 in purple and mode 5 in blue), corner state (mode 11 in purple) and bulk state (mode 10 in orange).

The plasmonic clusters are so small that it seems reasonable to treat it as a thin plate of continuous material by using effective-medium theory. However, as the cluster of nanoparticles is just one layer thick and the eigenfield extends into the vacuum, it is not so easy to define a thickness of the effective medium plate for the sake of field averaging when we do the effective medium parameter extraction. In order to understand qualitatively the formation of this kind of edge state, we consider coupled-dipole equations that can describe the response of these meta-arrays and obtain the eigenvalues of the corresponding response matrix and their mode distributions. Dipole approximation with quasi-static approximation in the tight binding model is applied to find the Hamiltonians. The Eqs. (1) and (2) represent the dipole moment ***P***_*n*_ at ***R***_*n*_ of a disk and dipole–dipole interaction coefficient *G*^0^(***R***_*nm*_) ***P***_*m*_, respectively [76]

(1) $$P_{n}=\alpha_{E}\underset{m\neq n}{\sum }G^{0}\left(R_{n}-R_{m}\right)P_{m}$$

(2) $$G^{0}\left(R_{nm}\right)P_{m}=\frac{1}{{R_{nm}}^{3}}\left[3{\hat{R}}_{nm}\left({\hat{R}}_{nm}\cdot P_{m}\right)-P_{m}\right]$$

where the *α*_*E*_ is an electric polarizability of the disks as *α*_*E*_ = 4*πɛ*_0_*r*^3^ (*ɛ* − *ɛ*_*g*_)/(*ɛ* + 2*ɛ*_*g*_). The *ɛ*_*g*_ is the background permittivity as 1, and the *ɛ* is the permittivity of disks calculated by the Drude model with simulated plasmonic frequency *ω*_p_ of 2*π* × 4.16*e*14 rad/s. *R*_*nm*_ is a magnitude of ***R***_*nm*_ and ${\hat{R}}_{nm}$ is a unit row vector of ***R***_*nm*_ [77]. We consider not only the nearest-neighbor interaction, but also the total electric fields induced by other dipoles, and obtain the energy eigenvalues in Figure 3 of C4R4 (purples) and C5R4 (blues and oranges) meta-arrays under XP and YP excitation. Detailed description can be found in the SM III. We further show the normalized mode distribution of different eigenmodes including the edge mode. The mode 9 in purple represents the edge state and the mode11 in purple represents the corner state in C4R4 meta-arrays under XP or YP excitation. The mode 5 in blue represents the edge state in C5R4 meta-arrays under XP excitation and the mode 10 in orange represents the bulk state in C5R4 meta-arrays under YP excitation. They are all consistent with experimental near-field images and simulated mode distributions. We note that the calculations can be performed without the quasi-static approximation. In that case, the mode distributions will not be affected, but the imaginary part of the eigenvalues will emerge which is correlated with the radiative widths that appear in the spectra. Since this work focuses on mode distribution, we therefore ignore the radiation loss and the retardation effect and use the quasi-static approximation.

Through the above analysis, we see that the experimentally observed edge state is one of the eigenmodes in this meta-array. Such edge modes are ubiquitous in small clusters that can described by dipolar interaction. Indeed, we found numerically that the existence of this kind of edge states does not depend that much on the material of the disk. For instance, when the metallic gold is replaced by the dielectric silicon, the edge states are still found to exist. In addition, there are lots of other eigenmodes in both simulated and calculated results, which their resonant wavelengths are near that in the edge mode. However, during the experimental measurements, the only observable mode is the edge mode through tuning the excitation laser wavelength from 690 to 900 nm. We further analyze this phenomenon and find that the edge-state mode distribution dominates the plasmonic resonance due to the normal incident excitation, and the other modes are difficult to excite under this mode of excitation. Therefore, the generation of the expected edge modes can be controlled by properly coupling them to the specific form of the external incident light. This is also verified by FDTD simulation which can refer to SM IV, and it is found that the edge-mode near-field intensity is much greater than that of other eigenmodes under the normal incident excitation. In the following section, we will give quantitative limiting parameters for the generation of edge states.

## 3 Influence of the structural details of meta-arrays on edge states

**Figure 4:**
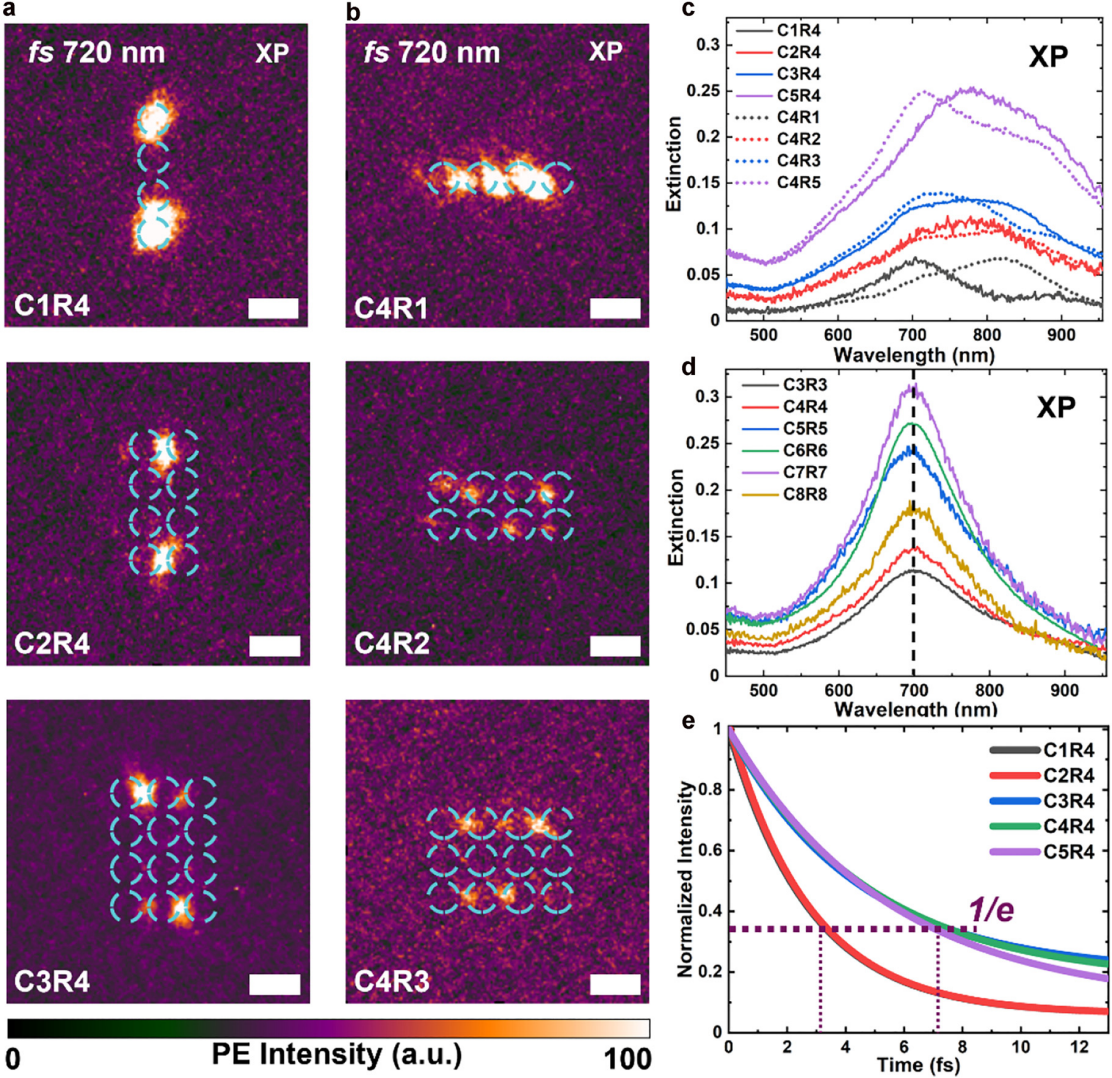
Near-field images and far-field spectra of plasmonic meta-arrays with different numbers of column and row. (a, b) Columns-dependence and rows-dependence of the near-field mode distribution of meta-arrays under XP excitation. The scale bar represented by a short white line in each figure represents 200 nm. The dashed cyan circles represent the spatial location of meta-arrays. The excitation wavelength is 720 nm at their peak wavelength. (c) Columns-dependence and rows-dependence of extinction in square lattices under XP excitation with wavelength range from 450 nm to 955 nm, marked by solid color lines and dashed color lines respectively. (d) Size-dependence of extinction in square lattices under XP light with wavelength range from 450 nm to 955 nm. The dashed black line indicates the single peak wavelength around 700 nm. (e) Simulated fitting curves of the dephasing time of each edge state in square-lattice meta-arrays with increasing column number from C1 to C5 while keeping the row number at 4. The dashed claret lines indicate the values of dephasing times.

Though this kind of edge state can easily be found in small clusters, there are still some influencing factors that will determine the details. We choose some examples to analyze the influence of column and row numbers, as well as the interparticle gap size, on the edge states in the near and far fields by experiments. Three different meta-arrays with the same row number of R4 and different column numbers from C1 to C3 are fabricated by EBL and measured by using PEEM instruments as shown in the Figure 4(a). The scale bars represent 200 nm. As the C4R4 and C5R4 arrays have been analyzed above, we only show the near-field images of C1R4, C2R4, and C3R4 meta-arrays under XP excitation. It is found that when the column number is 1, there is still edge state in the near field with the resonant peak wavelength of 720 nm, and its far-field extinction has one resonant peak wavelength of around 720 nm, which corresponds to the transverse mode of plasmonic resonance. With we increase the column number, the edge-states resonant peak wavelengths of different meta-arrays in the near field does not change and they stay at 720 nm. However, if the column number is larger than 1, there will be two peaks in the far-field extinction spectra with the meta-arrays from C2R4 to C5R4, as shown in Figure 4(c) with solid color lines. In addition, we simulated the change on the dephasing time of edge states as the column number increases in Figure 4(e). The dephasing time here refers to amplitude intensity decay of plasmonic modes. We monitored the curves of decay near the edge, fitted the curves and get the simulated dephasing time of the edge states. The results show that the dephasing time will gradually increase, and a sharp change will occur between the column number of 2 and 3, and then it reaches saturation. Increasing column number has little effect on the dephasing time after the column number is more than 3. Further increasing the size of metal meat-arrays can bring higher localized intensity of edge states, at the expense of a higher loss of the system. From the perspective of mode hybridization, when the column number is one or two, we observe simple dipole mode of longitudinal or transverse mode between disks; while when the column number is more than 3, high-order coupled modes will appear and this cause the increase of the dephasing time. Moreover, the suppression ratio values are obtained as 3.21, 3.76, and 6.01 from experimental results, and 3.38, 4.03, and 14.36 from FDTD simulation results for C1R4, C2R4, and C3R4 meta-arrays. The details can refer to SM V.

By rotating the sample 90°, different row numbers from R1 to R3 of meta-arrays with the same column number of 4 can be obtained. Under the same XP excitation, the experimental near-field results are shown in the Figure 4(b), and the scale bars represent 200 nm. In the case of C4R1 and C4R2 meta-arrays, there is no edge state but we observe excitations in the interior at the resonant peak wavelength of 720 nm. In the case of C4R3 and C4R4 (Figure 2(b)), the edge states reappear at the resonant peak wavelength of 720 nm. However, in the case of C4R5 (Figure 2(g)), there is also no edge state but bulk states at the resonant peak wavelength of 720 nm. This shows that the row number largely determines whether there can be edge states or not. The far-field extinction spectra are also shown in the Figure 4(c) with dashed color lines, and there are always two peaks except the C4R4 meta-arrays (Figure 4(d)). Although there are two peaks in the spectra, the edge state only corresponds to the peak with the shorter wavelength. The other peak with longer wavelength corresponds to the bulk state that can couple with far field radiation. It is believed that the symmetry of the finite meta-arrays will affect the shape of the far-field spectra. If the meta-arrays have equal column and row numbers, the extinction spectra will show symmetrical distributions with only one peak shown in Figure 4(d), marked by dashed black line. All these experimental results are consistent with the simulation and theoretical calculation results.

Due to the small gaps of each disk of 20 nm in the experiments, we further study the gap-size influence on the generation of the edge state. The gap size here refers to the distance from disk boundary to disk boundary. We use C4R4 meta-arrays as an example to analyze, and change the gap size as 10 nm, 30 nm, 50 nm, and 80 nm by FDTD simulations. Although edge states still exist, the suppression ratio decreases as 50.31, 5.09, 2.75, and 1.92. In addition, the simulated edge-state dephasing time also gradually decrease with the increase of gap size as shown in SM V. However, when we continue to increase the gap size to 120 nm (now the lattice constant is 240 nm, twice of the disk diameter size), the suppression ratio decreases to 1.35, which means that the edge state is not well defined. Gap size influences the coupling strength between disks. The results indicate that it requires strong near-field coupling strength to generate the edge state, and the coupling strength of disks is an important factor. From the above analysis, we can conclude that edge eigenmodes can exist in small meta-arrays with a strong coupling between the plasmonic particles in the array.

## 4 Edge states in different lattice types

**Figure 5:**
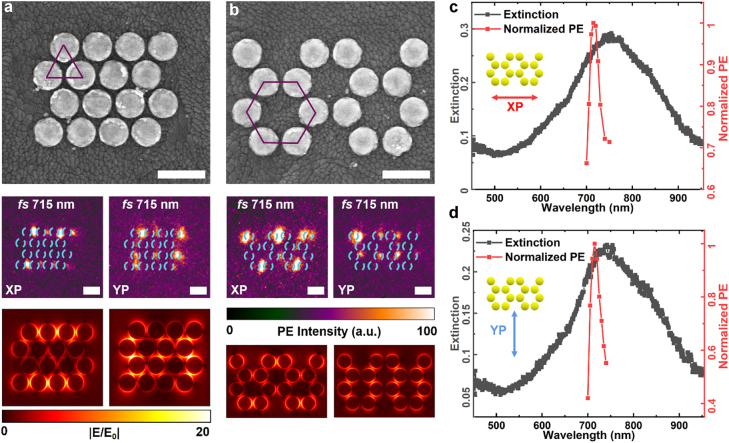
Experimental and simulated results of triangular and honeycomb lattice. (a, b) SEM images of the plasmonic meta-arrays with triangular and honeycomb lattices. Experimental near-field images and simulated electric field distributions are shown for XP (left panels) and YP (right panels) excitations. The excitation wavelengths are 715 nm at their peak wavelength, and the scale bar represented by a short white line in each figure represents 200 nm. (c, d) Spectra of far field and near field under XP and YP excitation in the honeycomb lattice respectively. The wavelength range of the figures is from 450 nm to 955 nm. The red two-way arrow indicates the XP light and the blue two-way arrow indicates the YP light.

Edge states in different symmetric lattices are also measured and it is verified that the edge states are indeed ubiquitous, independent of details. We fabricated triangular and honeycomb-lattice meta-arrays with four rows and each row has four gold disks. The diameter of each disk remains 120 nm and the lattice constant remains 140 nm with the nearest gap size of 20 nm. As shown in Figure 5(a), (b), the SEM images, experimental near-field images and simulated electric field distributions under XP and YP excitation are demonstrated. The excitation wavelength is 715 nm at the resonant peak wavelength, and the scale bars represent 200 nm with the experimental field of view of 5 um. The experimental images show that edge states are observed under XP excitation independent of whether the underlying structures are triangular or honeycomb. The suppression ratios in experimental results are 9.0 in triangular lattice and 7.28 in honeycomb lattice, and the simulated ratios are 10.35 and 8.72, respectively. While under YP light excitation with same wavelength, we observed the responses in the interior instead of edges. The edge states will appear at a longer wavelength of about 900 nm according to the FDTD simulations under YP excitation. However, as a result of the limitation of experimental excitation-laser central wavelength, the excitation power cannot reach the requirements and the field-enhancement intensity of the edge state under YP excitation is low, the edge states cannot be observed in experiments. The spectra of far field and near field under XP and YP excitation in honeycomb lattice are given in Figure 5(c), (d), respectively. It shows that in the far field, the peak wavelength under YP excitation is a little red shift than the peak wavelength under XP excitation, and in the near field, the two peak wavelengths keep the same. Due to the appearance of the similar edge states in different lattice types as that in square lattice, it illustrates that the edge states of eigenmode are generally applicable under polarization steering.

## 5 Generation of quantum entanglement in the meta-arrays

**Figure 6:**
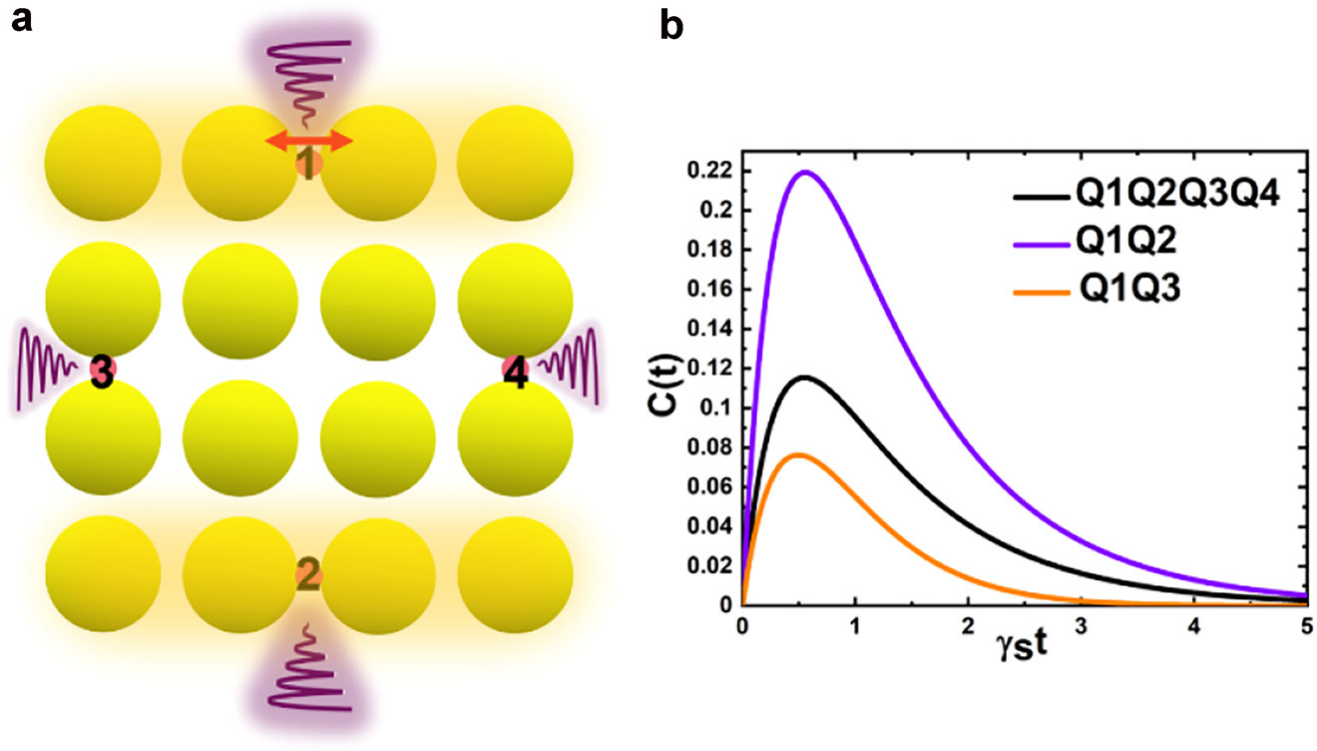
Schematic of the generation of quantum entanglement source. (a) Schematic diagram of the quantum entanglement with XP-dipole light. Four quantum dots are put in the middle of the four edges and number by Q1, Q2, Q3 and Q4 respectively. (b). Calculated total concurrence function (black), the concurrence function between Q1 and Q2 (purple) and the concurrence function between Q1 and Q3 (orange).

In addition, we find by simulation that the existence of the edge states is not compromised by structural modification such as missing disks or added obstacles on the edge, which indicates that this kind of edge states are robust to structural perturbation. The detailed results are in the SM VI. To go a step further, we consider that this unique edge states can help protect the correlation or entanglement of quantum pairs as well. Here we show a 4-qubit quantum entanglement. Through FDTD simulations, we put one dipole on the top row with XP light in the C4R4 meta-arrays to excite the edge state, and set the qubits at the middle of the top and bottom row, far left and right column. The qubits Q1, Q2, Q3, and Q4 are numbered as shown in Figure 6(a). Consider each qubit has two states, and *|eggg* > represents the state with first qubit is excited and others are at ground state. The rest can be done in the same manner. The dynamic evolution of the system is determined by the master equation:

(3) $$\begin{array}{ll} \frac{\partial \rho }{\partial t} & =-i\omega_{0}\underset{j}{\sum }\left[S_{j}^{z},\rho \right]-i\underset{i,j}{\sum }\Omega_{ij}\left[S_{i}^{+}S_{j}^{-},\rho \right]\\ & \;-\underset{i,j}{\sum }\gamma_{ij}\left[S_{i}^{+}S_{j}^{-}\rho -2S_{j}^{-}\rho S_{i}^{+}+\rho S_{i}^{+}S_{j}^{-}\right] \end{array}$$

where *i*, *j* = 1, 2, 3, 4. *ω*_0_ is the transition frequency of all qubits. *ρ* is the density matrix. $S_{i}^{+}=|e_{i}><g_{i}|,S_{i}^{-}=|ge_{i}|$ are the ladder operators. $S_{i}^{z}=1/2\;\left(\left|e_{i}><e_{i}|-|g_{i}><g_{i}\right|\right)$ is the z-component of 1/2-pseudospin operators. Then we can obtain the coupling coefficient between qubits with imaginary part *γ*_*ij*_ and the negative real part Ω_*ij*_.

(4) $$\gamma_{ij}=\frac{1}{\varepsilon_{0}}Im\left(\frac{\omega_{0}^{2}}{c^{2}\hbar }p_{i}\cdot G\left(r_{i},r_{j},\omega_{0}\right)\cdot p_{j}^{*}\right)$$

(5) $$\Omega_{ij}=-\frac{1}{\varepsilon_{0}}Re\left(\frac{\omega_{0}^{2}}{c^{2}\hbar }p_{i}\cdot G\left(r_{i},r_{j},\omega_{0}\right)\cdot p_{j}^{*}\right)$$

where the ***p*** is the dipole moment and $G\left(r_{i},r_{j},\omega_{0}\right)$ is the Green function,

(6) $$\left[\nabla \times \nabla \times -\frac{\omega_{0}^{2}}{c^{2}}\varepsilon \left(r_{i},\omega_{0}\right)\right]G\left(r_{i},r_{j},\omega_{0}\right)=\delta \left(r_{i}-r_{j}\right)$$

To quantitative describe the entanglement between the 4 qubits, we calculate the concurrence function. The detailed derivation process is in the SM VII, and the calculated results of concurrence function *C*(*t*) is shown in Figure 6(b). Since the maximum value of the concurrence function is more than 0.1 instead of zero, it proves that the 4-qubits have entanglement. Moreover, the entanglement degree between Q1 and Q2 is larger than the entanglement degree between Q1 and Q3 from Figure 6(b). The emission of light from an excited qubit on the edge row can be coupled far away to the qubit on the opposite-edge row due to the edge-state properties of these meta-arrays. The quantum entanglement generation through edge states implies that our platform has potential in the research of quantum information and quantum communication.

## 6 Conclusions

In this work, we showed that eigenmodes that are localized on the edges are ubiquitous in small plasmonic clusters, and these edge modes can be selectively excited by choosing the polarization of the incident field. This kind of plasmonic arrays can be very compact with a lattice of just a few rows and columns. They are small enough so that the bulk-edge correspondence of topological band theory is irrelevant but are big enough to distinguish between boundary and interior excitation; and they are macroscopic in the sense that classical electromagnetism provides an adequate description of the mode localization. When the normal incident light wavelength corresponds to the plasmonic resonant wavelength, the edge states will be excited, with strong field localization in the gaps of the edge particles. The small lattice numbers and narrow gap sizes are required to support the high suppression-ratio edge states, and which edge states will be excited depend on the incident polarization direction. We found that the excited edge states decay quickly into the interior as the gap sizes increase. We also experimentally found that the row number and the column number of the meta-arrays have influence on the edge-states and their excitation. In addition, the edge states are found to exist in different lattice types with small row and column numbers. Numerical simulations show that the edge states can generate and protect the quantum entanglement because of the robustness.

The small plasmonic clusters can be applied to many photonic devices with different functions. Due to the compact size and strong field distribution at the edges of the plasmonic systems, it can be used as ultrasmall light source emitters. For example, by covering the present plasmon configuration with nonlinear materials to achieve nonlinear modulation, nonlinear light sources or entangled quantum light sources can be obtained. Since the configuration is robust, the light sources will be efficient. Moreover, it can also be used as a high-precision plasmonic antenna, which can help send and receive signals. Similarly, by using the polarization multiplexing characteristics of the structures, multichannel ultrafast response optical switches or triggers in integrated optical chips can be realized. Different polarized lights can control the switching conditions of different channels. Furthermore, if the edge states of the configurations are carefully designed and connected to other edge states in different sites, the optical logic operation functions are realizable. In addition, because the interior excitations of the structures are suppressed and the boundaries are excited, it can also be used as a device protector. As long as this small structure is added where the device would not like to be excited by the pump light, it can ensure that the device will not respond to the external light field. This work provides a very simple way to realize edge states and can promote the applications of plasmonics in areas where energy and wave localization are useful, and has the potential in the future research of integrated optics from classical to quantum systems.

## 7 Methods

### 7.1 Simulation

We used commercial software (Lumerical FDTD solutions) to simulate the far-field and near-field characteristics of plasmonic meta-arrays with finite-difference time-domain (FDTD) method. The optical properties of Au in meta-arrays were obtained from Johnson-Christy model and the refractive index of ITO was set to 1.6. In the simulation model, the ITO was set as substrate, and the diameter of Au nanoparticle was 120 nm with the height of 30 nm. The plane wave excited the simulation region with linear polarization and the simulation region was meshed uniformly a 3 nm mesh size with periodic boundary conditions. We also used commercial software (MATLAB R2015a) to calculate the mode eigenvalues of different meta-arrays under the tight-binding model.

### 7.2 Fabrication and characterization of plasmonic meta-arrays

We cleaned the 150 nm-ITO-coated silica glass substrates by sequential ultrasonication for 5 min in acetone, methanol, and ultrapure water. A copolymer resist (ZEP520A, Zeon Chemicals) diluted with a ZEP thinner (1:1) is spin coated onto the substrates at 1000 rpm for 10 s and 4000 rpm for 60 s, and the substrates are prebaked at 180 °C for 2 min. The wet etching process is used for fabricating the nanopatterns followed by electron-beam lithography (ELS-F125, Elionix) with an electron current of 50 pA at 125 kV, developing with ZED-N50 for 48 s, depositing titanium (Ti) of 2 nm and Au film of 30 nm by helicon sputtering (MPS-4000C1/HC1, ULVAC), and lift-off with ZDMAC and cleaning by ultrasonication. After fabrication, photonic multichannel analyzer (PMA C7473, Hamamatsu Photonics) equipped with an optical microscope (BX-51 (Olympus)) is used for measuring the far-field spectra and the field-emission scanning electron microscopy (Helios NanoLab 600i) is used for observing the surface morphology of nanopatterns.

### 7.3 PEEM measurement

The PEEM measurements were performed with a PEEM microscope (AC-PEEM III, Elmitec GmbH) equipped with one UV light source (cutoff energy ∼4.9 eV) and two laser sources. One laser source (Tsunami, Spectra-Physics) has pulse duration of approximately 100 fs, is wavelength tunable from 690 to 1040 nm at a repetition rate of 77 MHz, and was used for wavelength-dependent PEEM measurements. Both lasers illuminated the sample surface at normal incidence, and a *λ*/2 waveplate was used to control the laser polarization. We measured the plasmonic near-field mode distribution under linear polarized incident light.

## Supporting information

Additional simulated and experimental results, including the suppression ratio, different mode-distribution intensity, dephasing time, and the robustness of the meta-arrays. Calculated results, including the methods of solving the eigenvalues and the entangled quantum generation (PDF).

## Supplementary Material

Supplementary Material Details

## References

[1] Ozawa T., Price H. M., Amo A. (2019). Topological photonics. Rev. Mod. Phys..

[2] Lu L., Joannopoulos J. D., Soljacic M. (2014). Topological photonics. Nat. Photon..

[3] Yan Q. C., Hu X. Y., Fu Y. L. (2021). Quantum topological photonics. Adv. Opt. Mater..

[4] Noh J., Benalcazar W. A., Huang S. (2018). Topological protection of photonic mid-gap defect modes. Nat. Photon..

[5] Harari G., Bandres M. A., Lumer Y. (2018). Topological insulator laser: theory. Science.

[6] Shen H. T., Zhen B., Fu L. (2018). Topological band theory for non-hermitian Hamiltonians. Phys. Rev. Lett..

[7] Amelio I., Carusotto I. (2020). Theory of the coherence of topological lasers. Phys. Rev. X.

[8] Horsley S. A. R., Woolley M. (2021). Zero-refractive-index materials and topological photonics. Nat. Phys..

[9] Bandres M. A., Wittek S., Harari G. (2018). Topological insulator laser: experiments. Science.

[10] Klembt S., Harder T. H., Egorov O. A. (2018). Exciton-polariton topological insulator. Nature.

[11] Xue H. R., Yang Y. H., Gao F., Chong Y. D., Zhang B. L. (2019). Acoustic higher-order topological insulator on a kagome lattice. Nat. Mater..

[12] Liu W. J., Hwang M., Ji Z. R., Wang Y. H., Modi G., Agarwal R. (2020). Z(2) photonic topological insulators in the visible wavelength range for robust nanoscale photonics. Nano Lett..

[13] Yang Y. H., Yamagami Y., Yu X. B. (2020). Terahertz topological photonics for on-chip communication. Nat. Photon..

[14] Kruk S., Slobozhanyuk A., Denkova D. (2017). Edge states and topological phase transitions in chains of dielectric nanoparticles. Small.

[15] Gorlach M. A., Ni X., Smirnova D. A. (2018). Far-field probing of leaky topological states in all-dielectric metasurfaces. Nat. Commun..

[16] Sinev I. S., Mukhin I. S., Slobozhanyuk A. P. (2015). Mapping plasmonic topological states at the nanoscale. Nanoscale.

[17] Honari-Latifpour M., Yousefi L. (2019). Topological plasmonic edge states in a planar array of metallic nanoparticles. Nanophotonics.

[18] Pocock S. R., Huidobro P. A., Giannini V. (2019). Bulk-edge correspondence and long-range hopping in the topological plasmonic chain. Nanophotonics.

[19] Proctor M., Huidobro P. A., Maier S. A., Craster R. V., Makwana M. P. (2020). Manipulating topological valley modes in plasmonic metasurfaces. Nanophotonics.

[20] Yan Q. C., Cao E., Sun Q. (2021). Near-field imaging and time-domain dynamics of photonic topological edge states in plasmonic nanochains. Nano Lett..

[21] You J. W., Ma Q., Lan Z. H., Xiao Q., Panoiu N. C., Cui T. J. (2021). Reprogrammable plasmonic topological insulators with ultrafast control. Nat. Commun..

[22] Bisharat D. J., Sievenpiper D. F. (2019). Electromagnetic-dual metasurfaces for topological states along a 1D interface. Laser Photon. Rev..

[23] Song Q. H., Odeh M., Zuniga-Perez J., Kante B., Genevet P. (2021). Plasmonic topological metasurface by encircling an exceptional point. Science.

[24] Longhi S. (2018). Non-hermitian gauged topological laser arrays. Ann. Phys.-Berl..

[25] Mehrabad M. J., Foster A. P., Dost R. (2020). Chiral topological photonics with an embedded quantum emitter. Optica.

[26] Ota Y., Takata K., Ozawa T. (2020). Active topological photonics. Nanophotonics.

[27] Shao Z. K., Chen H. Z., Wang S. (2020). A high-performance topological bulk laser based on band-inversion-induced reflection. Nat. Nanotechnol..

[28] Zeng Y. Q., Chattopadhyay U., Zhu B. F. (2020). Electrically pumped topological laser with valley edge modes. Nature.

[29] Dikopoltsev A., Harder T. H., Lustig E. (2021). Topological insulator vertical-cavity laser array. Science.

[30] Kim M., Jacob Z., Rho J. (2020). Recent advances in 2D, 3D and higher-order topological photonics. Light-Sci. Appl..

[31] Khanikaev A. B., Shvets G. (2017). Two-dimensional topological photonics. Nat. Photon..

[32] Lustig E., Segev M. (2021). Topological photonics in synthetic dimensions. Adv. Opt. Photonics.

[33] Xue H. R., Jia D., Ge Y. (2021). Observation of dislocation-induced topological modes in a three-dimensional acoustic topological insulator. Phys. Rev. Lett..

[34] Kruk S., Poddubny A., Smirnova D. (2019). Nonlinear light generation in topological nanostructures. Nat. Nanotechnol..

[35] Smirnova D. A., Smirnov L. A., Leykam D., Kivshar Y. S. (2019). Topological edge states and gap solitons in the nonlinear Dirac model. Laser Photon. Rev..

[36] Lan Z. H., You J. W., Panoiu N. C. (2020). Nonlinear one-way edge-mode interactions for frequency mixing in topological photonic crystals. Phys. Rev. B.

[37] Smirnova D., Leykam D., Chong Y. D., Kivshar Y. (2020). Nonlinear topological photonics. Appl. Phys. Rev..

[38] Smirnova D., Kruk S., Leykam D., Melik-Gaykazyan E., Choi D. Y., Kivshar Y. (2019). Third-harmonic generation in photonic topological metasurfaces. Phys. Rev. Lett..

[39] Yao S. Y., Wang Z. (2018). Edge states and topological invariants of non-hermitian systems. Phys. Rev. Lett..

[40] Song F., Yao S. Y., Wang Z. (2019). Non-hermitian topological invariants in real space. Phys. Rev. Lett..

[41] Zhao H., Qiao X. D., Wu T. W., Midya B., Longhi S., Feng L. (2019). Non-Hermitian topological light steering. Science.

[42] Okuma N., Kawabata K., Shiozaki K., Sato M. (2020). Topological origin of non-hermitian skin effects. Phys. Rev. Lett..

[43] Wang Y. P., You J. W., Lan Z. H., Panoiu N. C. (2020). Topological valley plasmon transport in bilayer graphene metasurfaces for sensing applications. Opt. Lett..

[44] You J. W., Lan Z. H., Panoiu N. C. (2020). Four-wave mixing of topological edge plasmons in graphene metasurfaces. Sci. Adv..

[45] Lu Y. X., Chen Y. H. (2021). Topologically protected plasmon mode with ultrastrong field localization in a graphene-based metasurface. Opt. Express.

[46] Qiu P. P., Liang R., Qiu W. B. (2017). Topologically protected edge states in graphene plasmonic crystals. Opt. Express.

[47] Bauer C. A., Werner G. R., Cary J. R. (2008). Truncated photonic crystal cavities with optimized mode confinement. J. Appl. Phys..

[48] He L. J., Shen L. F., Deng X. H., Yuan K. (2019). One-way edge modes in truncated semiconductor photonic crystal at terahertz frequencies. J. Opt..

[49] Li Z., Ma H., Wu R. X., Wu G. Z. (2020). Tuning the chiral edge states and unidirectional transmission by surface morphology of gyromagnetic photonic crystal. J. Opt..

[50] Lu J., Shen L. F., Deng X. H., Li X. E., Zheng X. D. (2013). Impact of photonic crystal boundary shape on the existence of one-way edge mode. Appl. Optics.

[51] Morrison S. K., Kivshar Y. S. (2006). Tamm states and nonlinear surface modes in photonic crystals. Opt. Commun..

[52] Vlasov Y. A., Moll N., McNab S. J. (2004). Observation of surface states in a truncated photonic crystal slab. Opt. Lett..

[53] Yang J. K., Kim S. H., Kim G. H., Park H. G., Lee Y. H., Kim S. B. (2004). Slab-edge modes in two-dimensional photonic crystals. Appl. Phys. Lett..

[54] Saito H., Mizuma S., Yamamoto N. (2015). Confinement of surface plasmon polaritons by heterostructures of plasmonic crystals. Nano Lett..

[55] Wu J. J., Wu F., Xue C. H. (2019). Wide-angle ultrasensitive biosensors based on edge states in heterostructures containing hyperbolic metamaterials. Opt. Express.

[56] Yan Z. B. (2019). Majorana corner and hinge modes in second-order topological insulator/superconductor heterostructures. Phys. Rev. B.

[57] Ling C. W., Xiao M., Chan C. T., Yu S. F., Fung K. H. (2015). Topological edge plasmon modes between diatomic chains of plasmonic nanoparticles. Opt. Express.

[58] Zhang S., Ye Z. L., Wang Y. (2012). Anti-hermitian plasmon coupling of an array of gold thin-film antennas for controlling light at the nanoscale. Phys. Rev. Lett..

[59] Wang W. J., Ramezani M., Vakevainen A. I., Torma P., Rivas J. G., Odom T. W. (2018). The rich photonic world of plasmonic nanoparticle arrays. Mater. Today.

[60] Kasani S., Curtin K., Wu N. Q. (2019). A review of 2D and 3D plasmonic nanostructure array patterns: fabrication, light management and sensing applications. Nanophotonics.

[61] Yang K., Yao X., Liu B. W., Ren B. (2021). Metallic plasmonic array structures: principles, fabrications, properties, and applications. Adv. Mater..

[62] Shrestha V. R., Lee S. S., Kim E. S., Choi D. Y. (2014). Aluminum plasmonics based highly transmissive polarization-independent subtractive color filters exploiting a nanopatch array. Nano Lett..

[63] Lin Y. H., Wang D. Q., Hu J. T. (2019). Engineering symmetry-breaking nanocrescent arrays for nanolasing. Adv. Funct. Mater..

[64] Wang D. Q., Wang W. J., Knudson M. P., Schatz G. C., Odom T. W. (2018). Structural engineering in plasmon nanolasers. Chem. Rev..

[65] Wang D. Q., Yang A. K., Wang W. J. (2017). Band-edge engineering for controlled multi-modal nanolasing in plasmonic superlattices. Nat. Nanotechnol..

[66] Yang A. K., Hoang T. B., Dridi M. (2015). Real-time tunable lasing from plasmonic nanocavity arrays. Nat. Commun..

[67] Yang A., Hryn A. J., Bourgeois M. R. (2016). Programmable and reversible plasmon mode engineering. P. Natl. Acad. Sci. USA.

[68] Liu J. X., Wang W. J., Wang D. Q. (2019). Spatially defined molecular emitters coupled to plasmonic nanoparticle arrays. P. Natl. Acad. Sci. USA.

[69] Lozano G., Louwers D. J., Rodriguez S. R. K. (2013). Plasmonics for solid-state lighting: enhanced excitation and directional emission of highly efficient light sources. Light-Sci. Appl.

[70] Lee C., Tame M., Lim J., Lee J. (2012). Quantum plasmonics with a metal nanoparticle array. Phys. Rev. A.

[71] Xu D., Xiong X., Wu L. (2018). Quantum plasmonics: new opportunity in fundamental and applied photonics. Adv. Opt. Photonics.

[72] Bitton O., Gupta S. N., Haran G. (2019). Quantum dot plasmonics: from weak to strong coupling. Nanophotonics.

[73] Bogdanov S. I., Boltasseva A., Shalaev V. M. (2019). Overcoming quantum decoherence with plasmonics. Science.

[74] Zhou Z. K., Liu J. F., Bao Y. J. (2019). Quantum plasmonics get applied. Prog. Quant. Electron.

[75] You C. L., Nellikka A. C., De Leon I., Magana-Loaiza O. S. (2020). Multiparticle quantum plasmonics. Nanophotonics.

[76] de Abajo F. J. G. (2007). Colloquium: light scattering by particle and hole arrays. Rev. Mod. Phys..

[77] Kim M., Rho J. (2020). Topological edge and corner states in a two-dimensional photonic Su-Schrieffer-Heeger lattice. Nanophotonics.

