# Supplementary material for: Edge states in plasmonic meta-arrays
Source: Nanophotonics. 2022 Jul 7;11(15):3495–507. doi: 10.1515/nanoph-2022-0258 (PMC11501916; doi:10.1515/nanoph-2022-0258)
Supplement: Supplementary file 1 — Supplementary Material Details [file j_nanoph-2022-0258_suppl.docx]

**Supplementary Materials**

Edge States in Plasmonic Meta-Arrays

**Contents**

1. Suppression ratio
2. Surface charge distributions of C5R4 meta-arrays
3. Eigenmodes
4. Comparison among mode distribution intensity under normal incident light via FDTD simulations and PEEM measurements
5. FDTD simulated suppression ratio and gap size-dependent dephasing time
6. Robustness of the meta-arrays
7. Method of the entangled quantum generation
8. **Suppression ratio**

We used commercial software (Lumerical FDTD solutions) to simulate the far-field and near-field characteristics of plasmonic meta-arrays with finite-difference time-domain (FDTD) method. The optical properties of Au in meta-arrays were obtained from Johnson-Christy model and the refractive index of ITO was set to 1.6. In the simulation model, the ITO was set as substrate, and the diameter of Au nanoparticle was 120nm with the height of 30nm. The plane wave excited the simulation region with linear polarization, and the simulation region was meshed uniformly a 3 nm mesh size with periodic boundary conditions.

In the main text, we define the suppression ratio as the ratio of the near-field enhancement density of the region produced by the edge units to the near-field enhancement density of the region produced by the bulk units. Here, we give more details as shown in Figure S1. S1a and b represent the selection method of simulation results and experimental results respectively. The selected regions are marked with rectangles and texts. We obtain the total intensity of each region with the same area and calculate the near-field energy density enhancement by dividing the total intensity by the area. Therefore, the suppression ratio is the edge-intensity density divided by the bulk-intensity density. Because the suppression ratio is used to describe the edge states of eigenmodes, we just need to ensure that the areas of the selected edge and bulk regions are the same. It is worth mentioning that the suppression ratios obtained from the experiments results are the average of multiple measurements.


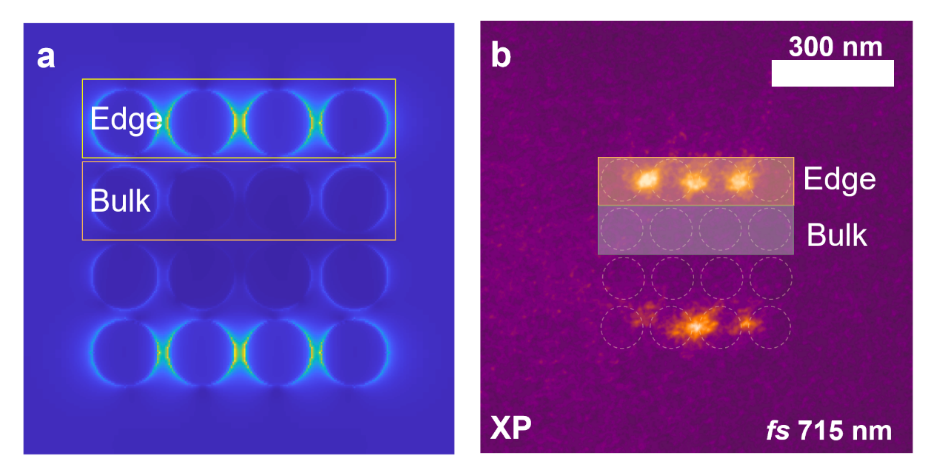


**Figure S1**. Schematic of the suppression ratio selection methods in FDTD simulations (**a**) and experimental results (**b**).

1. **Surface charge distributions in C5R4 meta-arrays**

We used commercial software (MATLAB R2015a) to calculate the mode eigenvalues of different meta-arrays under the tight-binding model. The surface charge distributions in C5R4 meta-arrays under XP and YP excitation are shown in Figure S2. S2a and S2b show the surface charge distributions of edge state with real and imaginary parts respectively, S2c and S2d show the surface charge distributions of bulk state with real and imaginary parts respectively. The dashed red rectangles indicate the high order mode of disks (S2b, c, d) or every four disks of the interior arrays composing a large quadrupole mode (S2a). The high order mode cannot be excited by the normal incident light; therefore, the near-field images present regular mode distributions. In addition, the real part and the imaginary part of the surface charge distributions both contribute the formation of the modes.


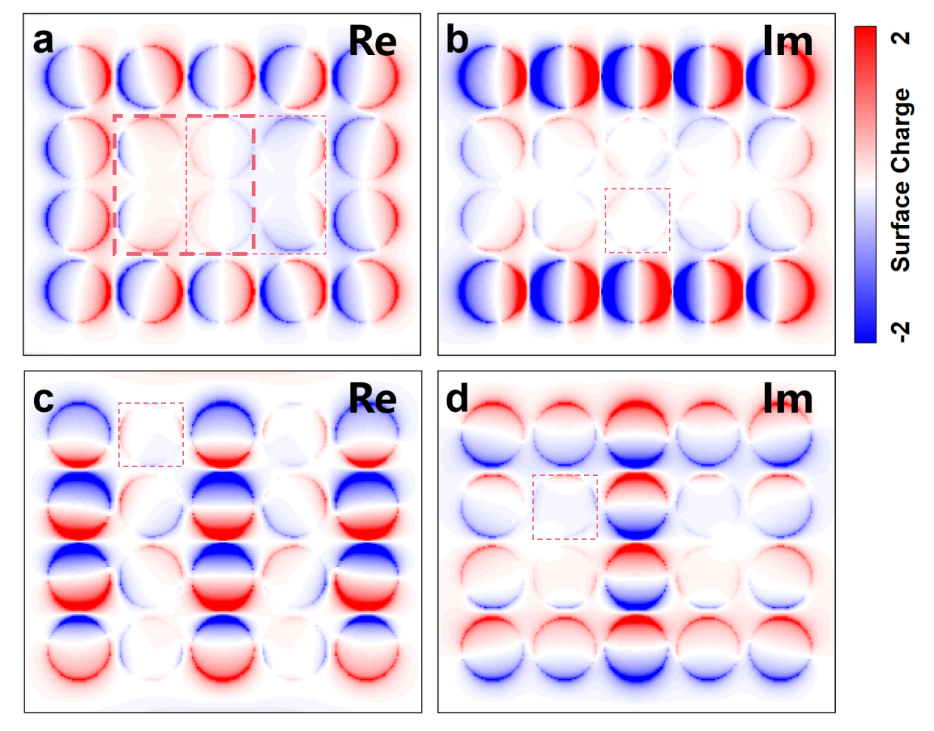


**Figure S2. a-b.** The surface charge distributions of C5R4 meta-arrays under XP excitation with real part (**a**) and imaginary part (**b**). **c-d.** The surface charge distributions of C5R4 meta-arrays under YP excitation with real part (**c**) and imaginary part (**d**).

1. **Calculation of the eigenmodes**

In order to calculate the eigenmodes of the meta-arrays, we use the dipole approximation and the quasi-static approximation and couple-dipole equations to write the Hamiltonians. The dipole approximation describes a nanoparticle as a point dipole, and usually applies to the radius of the nanoparticles *r* is very small in comparison to the separation *d* between the nanoparticles. In our system, we simplify the model by using dipole approximation to qualitatively illustrate the patterns of the eigenmodes, and we consider every disk as an excitable dipole mode. The quasi-static approximation helps us ignore the radiation loss and get real eigenvalues. The separation *d* is 140 nm and the excitation wavelength λ is 720 nm, which satisfies the requirements of inequality $d\ll\lambda$. In the original dipole-dipole interaction coefficient Equation (ES1), there are imaginary terms with wave vector *k*.

Due to the quasi-static approximation, and we ignore the terms with *k*, obtain the dipole-dipole interaction coefficient formation as the Equation (2) shows in the main text [1, 2].

$G^{0}(\boldsymbol{R}_{nm})\boldsymbol{P}_{m}=e^{ikr_{nm}}\left\{ \frac{k^{2}{\hat{\boldsymbol{r}}}_{nm}\times\boldsymbol{P}_{m}\times{\hat{\boldsymbol{r}}}_{nm}}{r_{nm}}+\frac{1-ikr_{nm}}{{r_{nm}}^{3}}\left[ 3{\hat{\boldsymbol{r}}}_{nm}\left( {\hat{\boldsymbol{r}}}_{nm}\cdot\boldsymbol{P}_{m} \right)-\boldsymbol{P}_{m} \right] \right\}$ (ES1)

We consider total electric fields induced by other dipoles on one site dipole and the off-diagonal terms of Hamiltonian are non-zero. Consequently, for example, the equation becomes a 32 × 32 matrix in a C4R4 meta-array, as each plasmonic disk has two degrees of freedom in the xy plane and the x- and y-polarized modes are not independent, and therefore the eigenvalue problem cannot be further decoupled. The results are shown in the main text of Figure 3, where the C5R4 meta-array has the same eigenvalues with different polarized light of XP and YP light.

1. **Comparison among mode distribution intensity under normal incident light via FDTD simulations and PEEM measurements**

According to the excitation method in the main text, the edge-state mode distribution dominates the plasmonic resonance within a certain wavelength range. However, other modes will also be excited with different intensity under normal incident light excitation as shown in Figure S3. The polarized direction of light keeps XP light. As the excitation wavelength is from 700 nm to 800 nm, there are always edge states where the upper and lower rows have electric field distribution and the interiors are suppressed. At the wavelength of 650 nm, the edge state is not obvious, and it gradually transits to the interior states as the electric field distribution shown in 600 nm. At the wavelength of 575 nm, it shows a corner state, while at the wavelength of 550 nm, it shows another edge states where the electric field distributions are localized at the left and the right columns. There are obvious mode intensity differences among different excited wavelengths.

In addition, the experimental results measured by PEEM instruments also demonstrate the intensity differences of the excitation wavelengths from 690 nm to 850 nm, as shown in Figure S4. The excited method is the same with the simulations. The main photoemission intensity distributions of the meta-array are marked by the yellow rectangles. Here, we demonstrate the near-field images with fs-laser and UV light, which can show the hot spots of plasmonics and the spatial positions of the disks at the same time. At the wavelength of 690 nm, it shows like another edge states where the electric field distributions are localized at the left and the right columns; at the wavelength of 720 nm, it shows the edge states mentioned in the main text; while at the wavelength of 850 nm, we can only see the intensity excited by the UV light without the plasmonics and the hot spots disappear, which shows the un-excited mode and corresponds to the simulated results. There are some differences between simulated and experimental results due to the fabrication errors and the scattered light from the background, while the conclusions are clear and identical.


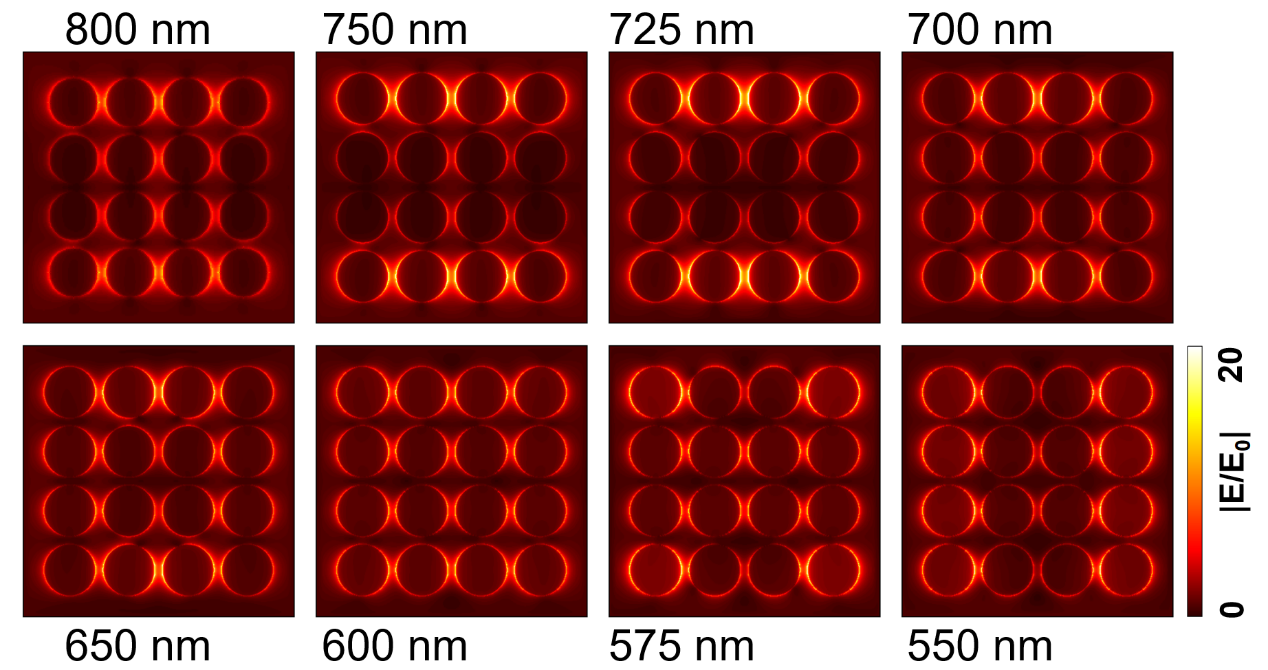


**S3.** Different modes’ distribution intensity under normal incident light excitation with different excitation wavelengths by using FDTD simulations.


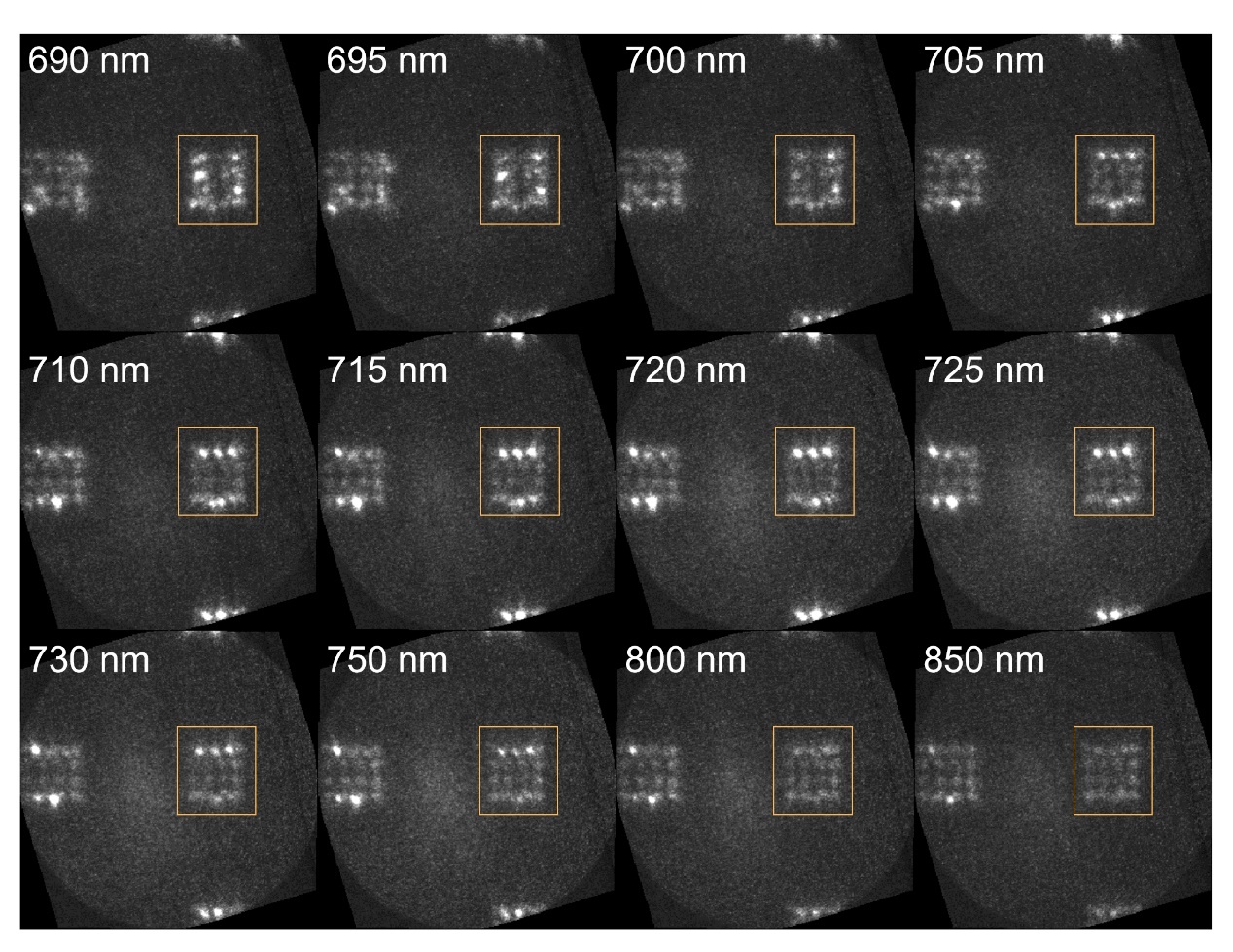


**S4.** The experimental results of the modes’ distribution intensity of the excitation wavelengths from 690 nm to 850 nm. The regions marked by the yellow rectangles represent the original measured modes of C4R4 meta-arrays under fs-laser and UV light at the same time.

1. **Simulated suppression ratio and gap size-dependent dephasing time**

We simulated the suppression ratio to compare with the experimental results as shown in Figure S5. S5a gives the simulated suppression ratio values of the C1R4, C2R4, C3R4, C4R4 and C5R4 meta-arrays. There is a sharp increase of the suppression ratio as the column number is from C2 to C3, which shows the same property as dephasing time. The inset of Figure S5a is the enlarged part of the dashed circled region. In addition, it is worth mentioning that the suppression-ratio peak wavelengths of meta-arrays with different column numbers have red shift as the column number increases, which is not found in the experimental results. The peak wavelength of the edge states keeps 720 nm with fs-laser in the PEEM measurements. We think that the difference comes from the complex high-order modes when the meta-arrays with large column number. Due to the limitation of the experiments, including the uniformity of sample fabrication and the normal incident light, high-order modes cannot be totally excited. While in the simulation, some high-order modes will be excited because of the ideal conditions, which leads to the red shift under the same XP light excitation.

The gap size also influences the suppression ratio of the edge states. Therefore, we also simulate the gap size-dependent suppression-ratio value, as well as the dephasing time of Figure S5b and S5c. Figure S5b shows that the suppression ratio will decrease quickly as the gap size increases. This is understandable because each particle should have the same excitation in the limit of large interparticle separation. When the suppression ratio is lower than 1.5 as the case of the gap size of 120 nm, no edge state can be meaningfully assigned. The inset of Figure S5b is the enlarged part of the dashed circled region. Their peak wavelength of the edge states mainly keeps unchanged when the gap size changes, except the case of the gap size with 10 nm. The narrow gap size indicates that there may have quantum effect which can lead to the difference. Figure S5c demonstrates the simulated dephasing time of the gap size dependence. The dephasing time also decreases as the gap size increases. The position marked by *1/e* represents the life time of the edge mode.


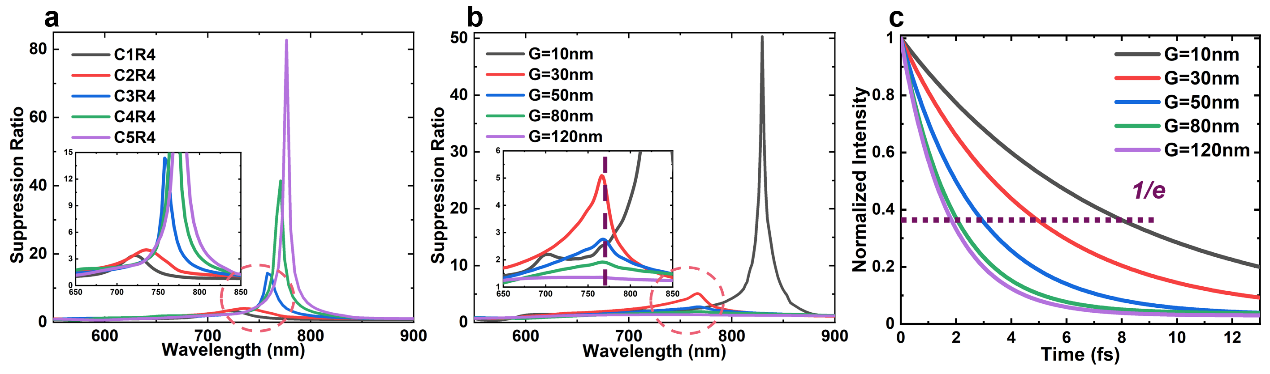


**S5**. **a.** Simulated suppression ratio values of the C1R4, C2R4, C3R4, C4R4 and C5R4 meta-arrays. **b.** Simulated suppression ratio values of the C4R4 meta-arrays with different gap sizes. **c.** Simulated dephasing times of the C4R4 meta-arrays with different gap sizes.

1. **Robustness of the meta-arrays**

The meta-arrays can support edge states that are resilient against perturbation. Figure S6 shows the simulated electric field distributions and the corresponding suppression ratios. S6a-b show the electric field distributions of the C4R4 meta-array with one disk missing. When the missing disk is in the interior of the meta-array, the suppression ratio is 41.81 shown in S6d in red line; when the missing disk is on the edge of the meta-array, the suppression ratio is 7.05 shown in S6d in black line. Though the suppression ratio of the edge-disk missing decreases quickly, there is still obvious edge state. S6c shows the electric field distributions of the triangular-lattice meta-array with one edge-disk missing, and the corresponding suppression ratio is 3.64 shown in S6d in blue lines. Because the suppression ratios are all higher than 1.5, we can consider that these edge states still exist and are robust.


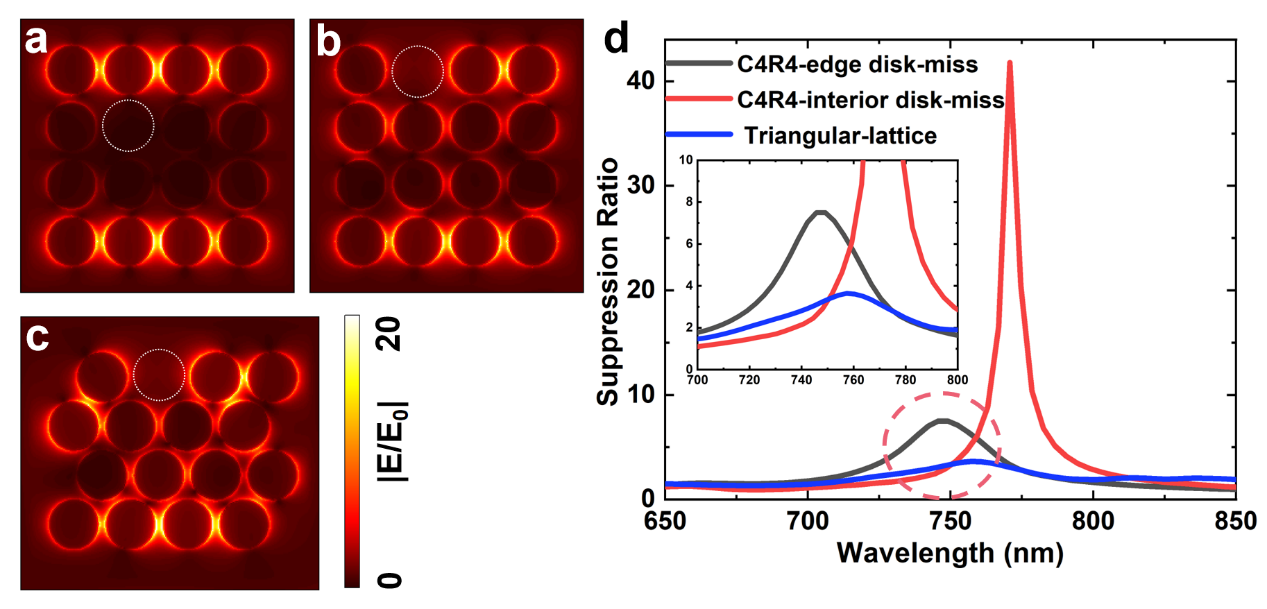


**S6. a.** Simulated electric field distributions of the C4R4 meta-array with one interior disk missing. **b.** Simulated electric field distributions of the C4R4 meta-array with one edge disk missing. **c.** Simulated electric field distributions of the triangular-lattice meta-array with one edge disk missing. **d.** Related suppression ratios in S6**a-c**.

1. **Method of the entangled quantum generation**

Here, we will show in detail that it can realize 4-qubit quantum entanglement. We set the four qubits at the middle of the top and bottom rows, left and right columns. Their z-positions are 0.015 μm. Consider each qubit has two states, $\left. |eggg \right\rangle$ represent the first qubit is excited and others are at ground state, the rest can be done in the same manner. The dynamic evolution of the system is determined by the master equation in the main text as Equation (3) shows. After the Green function is defined, we show the XP input light can excited the ‘row’ edge state, and YP input light excited ‘column’ edge state. Therefore, qubits 1,2 are set to be x-polarization and 3,4 are y-polarization. Then, we define the following symbols from Equation ES2 to ES7 [3,4].

$\Omega_{s}=\Omega_{ii}=-\frac{1}{\varepsilon_{0}}\mathrm{Re}(\frac{\omega_{0}^{2}}{c^{2}\hbar}\boldsymbol{p}_{\boldsymbol{i}}\mathbb{\cdot G(}\boldsymbol{r}_{\boldsymbol{i}}\boldsymbol{,}\boldsymbol{r}_{\boldsymbol{i}},\omega_{0})\cdot\boldsymbol{p}_{\boldsymbol{i}}^{\boldsymbol{*}})$ (ES2)

$\gamma_{s}=\gamma_{ii}=\frac{1}{\varepsilon_{0}}\mathrm{Im}(\frac{\omega_{0}^{2}}{c^{2}\hbar}\boldsymbol{p}_{\boldsymbol{i}}\mathbb{\cdot G(}\boldsymbol{r}_{\boldsymbol{i}}\boldsymbol{,}\boldsymbol{r}_{\boldsymbol{i}},\omega_{0})\cdot\boldsymbol{p}_{\boldsymbol{i}}^{\boldsymbol{*}})$ (ES3)

$\Omega_{c1}=\Omega_{12}=\Omega_{34}=-\frac{1}{\varepsilon_{0}}\mathrm{Re}(\frac{\omega_{0}^{2}}{c^{2}\hbar}\boldsymbol{p}_{\boldsymbol{x}}\mathbb{\cdot G(}\boldsymbol{r}_{\boldsymbol{1}}\boldsymbol{,}\boldsymbol{r}_{\boldsymbol{2}},\omega_{0})\cdot\boldsymbol{p}_{\boldsymbol{x}}^{\boldsymbol{*}})$ (ES4)

$\gamma_{c1}=\gamma_{12}=\gamma_{34}=\frac{1}{\varepsilon_{0}}\mathrm{Im}(\frac{\omega_{0}^{2}}{c^{2}\hbar}\boldsymbol{p}_{\boldsymbol{x}}\mathbb{\cdot G(}\boldsymbol{r}_{\boldsymbol{i}}\boldsymbol{,}\boldsymbol{r}_{\boldsymbol{i}},\omega_{0})\cdot\boldsymbol{p}_{\boldsymbol{x}}^{\boldsymbol{*}})$ (ES5)

$\Omega_{c2}=\Omega_{13}=\Omega_{14}=\Omega_{24}=-\frac{1}{\varepsilon_{0}}\mathrm{Re}(\frac{\omega_{0}^{2}}{c^{2}\hbar}\boldsymbol{p}_{\boldsymbol{x}}\mathbb{\cdot G(}\boldsymbol{r}_{\boldsymbol{1}}\boldsymbol{,}\boldsymbol{r}_{\boldsymbol{3}},\omega_{0})\cdot\boldsymbol{p}_{\boldsymbol{y}}^{\boldsymbol{*}})$ (ES6)

$\gamma_{c2}=\gamma_{13}=\gamma_{14}=\gamma_{24}=\frac{1}{\varepsilon_{0}}\mathrm{Im}(\frac{\omega_{0}^{2}}{c^{2}\hbar}\boldsymbol{p}_{\boldsymbol{x}}\mathbb{\cdot G(}\boldsymbol{r}_{\boldsymbol{1}}\boldsymbol{,}\boldsymbol{r}_{\boldsymbol{3}},\omega_{0})\cdot\boldsymbol{p}_{\boldsymbol{y}}^{\boldsymbol{*}})$ (ES7)

The above derivation process considers the symmetry property of qubits. Moreover, we also have reciprocal relation $\Omega_{ij}=\Omega_{ji}, \gamma_{ij}=\gamma_{ji}$. All parameters can be calculated numerically by using FDTD simulations as shown in Equation from ES8 to ES11.

$\gamma_{c1}=0.5453\gamma_{s}$ (ES8)

$\Omega_{c1}=0.1545\gamma_{s}$ (ES9)

$\gamma_{c2}=0.0624\gamma_{s}$ (ES10)

$\Omega_{c2}=0.1989\gamma_{s}$ (ES11)

If we set the initial condition that the qubit 1 is excited while others are all at the ground state, the dynamical equation can be expressed as Equation ES12：

$\frac{\partial\rho_{\alpha\beta}}{\partial t}=i\left( \omega_{\alpha}^{*}-\omega_{\beta} \right)\rho_{\alpha\beta}$ (ES12)

where $\rho_{\alpha\beta}=\left\langle\alpha| \rho| \beta\right\rangle$($\alpha,\beta=a,b,c,d)$. By defining the following quantum states, we can calculate the concurrence function.

$W=\omega_{0}+\Omega_{s}-i\gamma_{s}, K_{1}=\Omega_{c1}-i\gamma_{C1}, K_{2}=\Omega_{c2}-i\gamma_{C2}$ (E13)

$|\left. a \right\rangle=\frac{1}{\sqrt{2}}[|\left. eggg \right\rangle-\left| \left. gegg \right\rangle\right] \omega_{a}=W-K_{1}$ (ES14)

$|\left. b \right\rangle=\frac{1}{\sqrt{2}}[|\left. ggeg \right\rangle-\left| \left. ggge \right\rangle\right] \omega_{b}=W-K_{1}$ (ES15)

$|\left. c \right\rangle=\frac{1}{\sqrt{2}}[|\left. eggg \right\rangle+\left| \left. gegg \right\rangle-|\left. ggeg \right\rangle-|\left. ggge \right\rangle\right] \omega_{c}=W+K_{1}-2K_{2}$ (ES16)

$|\left. d \right\rangle=\frac{1}{\sqrt{2}}[|\left. eggg \right\rangle+\left| \left. gegg \right\rangle+|\left. ggeg \right\rangle+|\left. ggge \right\rangle\right] \omega_{d}=W+K_{1}+2K_{2}$ (ES17)

Due to the initial condition, the density matrix elements which contains the term of 2 qubits excited vanish. To quantitative describe the entanglement between the 4 qubits, we calculate the concurrence function [5]. We have 6 choices of subsystem, and each choice (labeled $\alpha$) contains 2 qubits $i\mathrm{and}j(i,j=1,2,3,4)$. Define antilinear operator $\Theta_{\alpha}$ which satisfied the relation:

$\Theta_{\alpha}\left. |e_{i}e_{j} \right\rangle=\left. -|g_{i}g_{j} \right\rangle\Theta_{\alpha}\left. |g_{i}g_{j} \right\rangle=\left. -|e_{i}e_{j} \right\rangle$ (ES18)

$\Theta_{\alpha}\left. |e_{i}g_{j} \right\rangle=\left. |g_{i}e_{j} \right\rangle\Theta_{\alpha}\left. |g_{i}e_{j} \right\rangle=\left. |e_{i}g_{j} \right\rangle$ (ES19)

For pure state, the concurrence function of subsystem is as follows:

$C_{\alpha}\left( \Phi\right)=|\left\langle\Phi| \Theta_{\alpha} | \Phi\right\rangle|$ (ES20)

$C\left( \Phi\right)=\sqrt{\sum_{\alpha} C_{\alpha}^{2}\left( \Phi\right)}$ (ES21)

Suppose the density matrix can be decomposed to summation of pure state.

$\rho=\sum_{i} p_{i}\left\langle\Phi_{i} | \Phi_{i} \right\rangle$ (ES22)

$C=\sum_{i} p_{i}C_{i}\left( \Phi\right)$ (ES23)

As shown in Figure 6 in the main text, the maximum value of concurrence function is more than 0.1. Since the concurrence function is not equal to 0, it proves that the 4-qubits exist entanglement with each other.

**References:**

[1] Kim M, Rho J. Topological edge and corner states in a two-dimensional photonic Su-Schrieffer-Heeger lattice. *Nanophotonics.* **9**, 3227-3234 (2020).

[2] Li YL, Sun Q, Zu S, Shi X, Liu YQ, Hu XY*, et al.* Correlation between Near-Field Enhancement and Dephasing Time in Plasmonic Dimers. *Physical Review Letters* **124**, 163901 (2020).

[3] Gangaraj SAH, Hanson GW, Antezza M. Robust entanglement with three-dimensional nonreciprocal photonic topological insulators. *Phys Rev A* **95**, 063807 (2017).

[4] Biehs SA, Agarwal GS. Qubit entanglement across epsilon-near-zero media. *Phys Rev A* **96**, 022308 (2017).

[5] Wootters WK. Entanglement of Formation and Concurrence. *Quantum Inf Comput* **1**, 27-44 (2001).
